# Enhancement of Biodegradability of Chicken Manure via the Addition of Zeolite in a Two-Stage Dry Anaerobic Digestion Configuration

**DOI:** 10.3390/molecules29112568

**Published:** 2024-05-30

**Authors:** Achilleas Kalogiannis, Ioanna A. Vasiliadou, Athanasios Tsiamis, Ioannis Galiatsatos, Panagiota Stathopoulou, George Tsiamis, Katerina Stamatelatou

**Affiliations:** 1 Department of Environmental Engineering, Democritus University of Thrace, Vas. Sofias 12, GR-67132 Xanthi, Greece; achkalog@env.duth.gr (A.K.); ivasiliadou@uowm.gr (I.A.V.); 2 Department of Chemical Engineering, University of Western Macedonia, GR-50100 Kozani, Greece; 3 Laboratory of Systems Microbiology and Applied Genomics, Department of Sustainable Agriculture, University of Patras, GR-30131 Agrinio, Greece; etsiamis9@gmail.com (A.T.); jgalia96@gmail.com (I.G.); panstath@upatras.gr (P.S.); gtsiamis@upatras.gr (G.T.)

**Keywords:** chicken manure, biogas, zeolite, leach bed reactor, 16S amplicon sequencing

## Abstract

Leach bed reactors (LBRs) are dry anaerobic systems that can handle feedstocks with high solid content, like chicken manure, with minimal water addition. In this study, the chicken manure was mixed with zeolite, a novel addition, and packed in the LBR to improve biogas production. The resulting leachate was then processed in a continuous stirred tank reactor (CSTR), where most of the methane was produced. The supernatant of the CSTR was returned to the LBR. The batch mode operation of the LBR led to a varying methane production rate (MPR) with a peak in the beginning of each batch cycle when the leachate was rich in organic matter. Comparing the MPR in both systems, the peaks in the zeolite system were higher and more acute than in the control system, which was under stress, as indicated by the acetate accumulation at 2328 mg L^−1^. Moreover, the presence of zeolite in the LBR played a crucial role, increasing the overall methane yield from 0.142 (control experiment) to 0.171 NL CH_4_ per g of volatile solids of chicken manure entering the system at a solid retention time of 14 d. Zeolite also improved the stability of the system. The ammonia concentration increased gradually due to the little water entering the system and reached 3220 mg L^−1^ (control system) and 2730 mg L^−1^ (zeolite system) at the end of the experiment. It seems that zeolite favored the accumulation of the ammonia at a lower rate (14.0 mg L^−1^ d^−1^) compared to the control experiment (17.3 mg L^−1^ d^−1^). The microbial analysis of the CSTR fed on the leachate from the LBR amended with zeolite showed a higher relative abundance of *Methanosaeta* (83.6%) compared to the control experiment (69.1%). Both CSTRs established significantly different bacterial profiles from the inoculum after 120 days of operation (*p* < 0.05). Regarding the archaeal communities, there were no significant statistical differences between the CSTRs and the inoculum (*p* > 0.05).

## 1. Introduction

Chicken meat production is undeniably the most significant portion of total meat production. According to [1], chicken meat has been experiencing the most significant growth since 2000, accounting for 35% of global production in 2019 (118 out of 337 million tonnes of total meat). Recent estimates (2022) indicate that poultry meat production has increased, reaching 136 million tonnes [2]. The shift in meat consumption towards chicken meat is mainly due to its lower price, convenient use for food preparation, and the perception that it is healthier [1].

In the EU 27, per capita consumption of chicken meat has remained relatively stable (23.4–23.7 kg/capita between 2019 and 2020), while production in absolute values reached a peak in 2020 (14 million tonnes), decreasing slightly in 2022 (13.5 million tonnes) [2]. It is crucial to meet the environmental, social, and economic pillars of sustainability, including animal welfare and the use of antibiotics, to ensure sustainability in the poultry meat sector while maintaining the industry’s resilience. It has been recognized that if strict measures are not adopted to fulfill the sustainability goals, the poultry industry will be severely impacted [2]. Therefore, all stakeholders must concentrate efforts to meet sustainability goals [3]. The excrement of chickens, which includes urine and feces, is produced in large quantities and is known as chicken manure (CM). This manure is mixed with bedding material, feathers, and leftover feed to form poultry litter when raising broiler chickens. The number of broiler chickens slaughtered for meat shows how much CM is produced. In 2022, approximately 6.6 billion, 11.7 billion, and 79.7 billion broiler chickens were slaughtered for meat in the EU-27, Europe, and worldwide, respectively. Based on the assumptions made by Tanczuk et al. [4]—65 g of CM produced per day per broiler, 42 days per breeding cycle, and six cycles per year—we can estimate that the amount of CM produced from broilers in 2022 will be around 18 million tons, 32 million tons, and 141 million tons in the EU-27, Europe, and the world, respectively.

The breeding of chickens in intensive farming facilities has significant environmental consequences, including greenhouse gas emissions like carbon dioxide, methane, ammonia, and nitrogen oxide produced during poultry production. The technology used to treat chicken manure also contributes to greenhouse gas emissions [5]. There are two main methods of treating chicken manure: biological and thermochemical. Biological processes, such as anaerobic digestion (AD) and composting, are environmentally friendly and require less energy [6]. AD is the most efficient method, producing the lowest emissions, especially if the digestate is kept in closed tanks. Furthermore, minimizing biogas leakage during storage and utilization is essential [5]. The anaerobic digestion process promotes sustainability by recovering the manure’s energy potential, reducing nitrous oxide formation, and increasing the nitrogen content in the digestate, making it more valuable for agriculture.

It is worth noting that the AD of CM is challenging due to the high content of total Kjendahl nitrogen (TKN) present, which ranges from 12.4 to 52.5 g kg^−1^ of total solids, with most of it consisting of ammonia nitrogen (AN) [7]. AN is beneficial at concentrations of 50–200 mg L^−1^ and has no adverse effects at 200–1000 mg L^−1^ concentrations. However, it can become inhibitory at concentrations between 1500 and 3000 mg L^−1^, especially at higher pH levels and temperatures. Concentrations exceeding 3000 mg L^−1^ become inhibitory at any pH level [8].

However, microorganisms have a wide adaptability range and can function properly even at a concentration of 11,800 mg L^−1^, provided they are adjusted accordingly. Microorganisms’ high tolerance to ammonia can also be attributed to metabolic changes and population shifts towards a syntrophic relationship between acetate-oxidizing bacteria and hydrogen-utilizing methanogens [8,9].

There are several methods available to deal with AN, including membrane separation [10], ammonia stripping [11], trace elements [12,13] or biochar [14] addition, and the use of zeolite as an adsorption agent [15], among others. Zeolite is a crystalline mineral that is efficient in ion exchange, making it suitable for removing ammonium and other cations [16]. The negative surface charge of zeolite allows for effective cation adsorption in addition to ion exchange. Its ability to retain ammonium while releasing other cations makes zeolite a potential enhancer of AD due to its ability to alleviate AN inhibition. However, zeolite has shown multiple benefits to anaerobic digestion (AD) consortiums beyond just ammonia retention. Simply reducing the size of ammonia in the system only partially explains the subsequent increase in methane yield (as per Fotidis et al. [15]). It has been suggested that the cations released once ammonium cations are removed, along with the microenvironment created by the zeolite, have a positive impact on the anaerobic microorganisms. Zeolites promote micro-organism immobilization and the formation of biofilms, which can help protect micro-organisms from the harsh conditions of the bulk environment.

Additionally, zeolites have been modified with NaOH treatment to enhance their capacity for the adsorption of ammonia and sulfate, which is another common inhibitor of AD [17]. This is particularly useful in digesters treating animal wastes and manure with high nitrogen and sulfate loads, leading to co-inhibition from ammonia and sulfate. Furthermore, the simultaneous application of zeolite and bischofite, a hydrous magnesium chloride mineral, to the AD of swine manure has significantly improved methane yield and struvite production [18]. The synergistic effect of zeolite combined with bischofite is due to the Mg^2+^ cations released upon NH^4+^ adsorption via ion exchange or the Mg^2+^ adsorption on the zeolite [19]).

This study is a follow-up to previous research on generating biogas from CM using leach bed reactors (LBR) combined with a methanogenic continuous stirred tank reactor. Since CM has a high total solids content, dry anaerobic digestion (AD) systems like LBR are ideal for its treatment [20]. LBRs can be used alone or in combination with other bioreactors, with the latter approach offering more benefits as it prevents inorganic material build-up in the second stage, where the methanogenic process occurs [20,21]. Previous research used zeolite as a bulking agent in a three-LBR and one-CSTR system, yielding 0.17 NL CH_4_ g^−1^ of Volatile Solids (VS) added. This study further evaluated the combination of LBR with CSTR by operating two systems in parallel, one using zeolite and the other using river pebbles (as an inert material without ion exchange properties compared to zeolite) of similar diameter and quantity as the control experiment. The two systems operated for 120 days, and microbial analysis showed the evolution of the CSTR microbiome from inoculation to the end of the experiment.

## 2. Results

### 2.1. Performance of the LBR-CSTR Systems

Two systems consisting of an LBR coupled with a CSTR were tested in parallel for 120 days. One system contained zeolite and the other pebbles as a control. Every 14 days, both LBRs were emptied and refilled with a fresh CM mixture containing zeolite or pebbles and wood chips, making the solid retention time (SRT) equal to 14 d. The SRT was close to 15 d, which was chosen in previous work [7], and was lower than in similar works with LBR in the literature. Each feeding indicated a new cycle, making the experiment a successive series of eight cycles. The leachate was removed approximately three days after feeding the LBR and fed to the Continuous Stirred Tank Reactor (CSTR). The removed leachate was replenished with the supernatant of the CSTR after stirring, which had been stopped for one hour to remove the effluent of the CSTR with as few solids as possible. Leachate removal from the LBR, leachate feeding to the CSTR, and CSTR effluent feeding to the LBR were repeated thrice a week.

**Figure 1:**
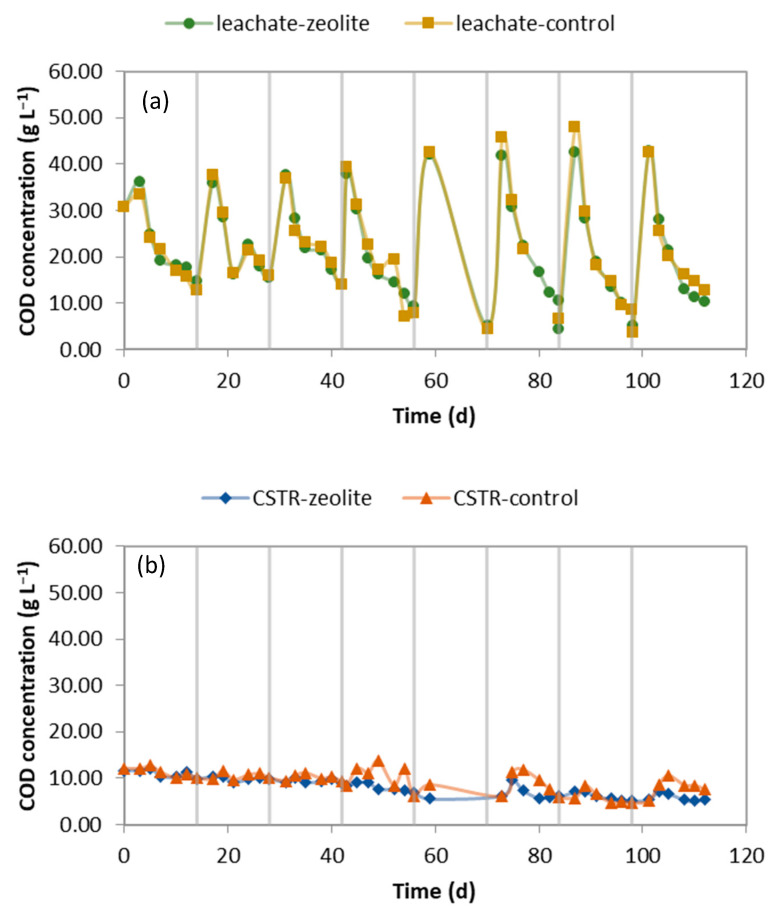
The COD concentration of (**a**) the leachate produced by the LBRs and (**b**) the CSTR effluent. The vertical lines indicate when the LBR was emptied and filled with fresh CM mixture.

**Figure 2:**
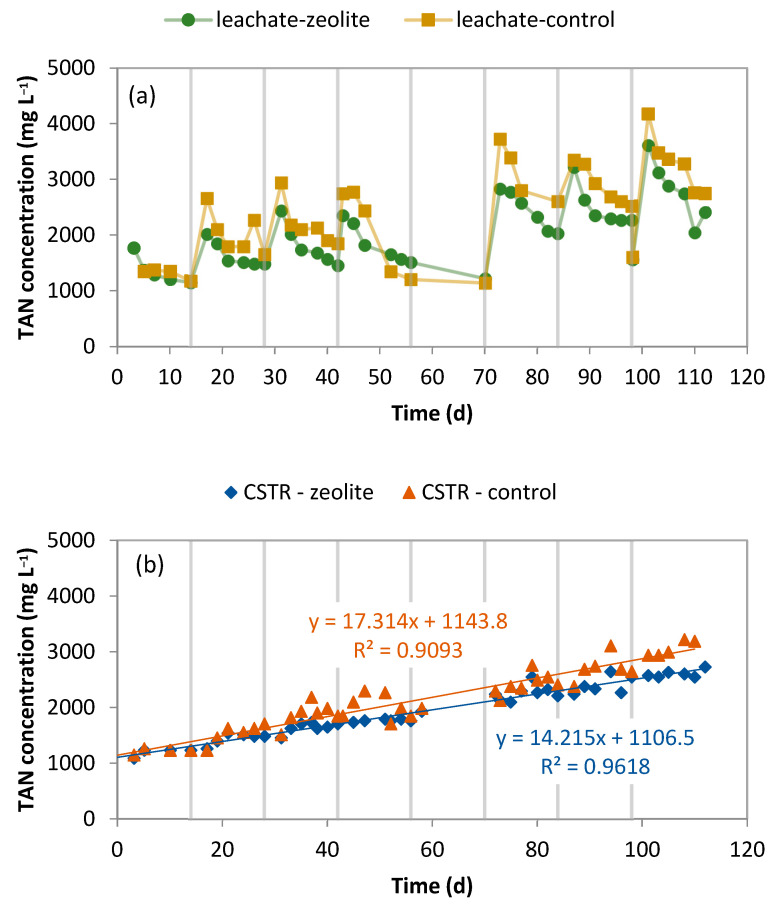
TAN concentration in (**a**) the leachate produced by the LBRs and (**b**) the CSTR effluent. The vertical lines indicate when the LBR was emptied and filled with fresh CM mixture.

The chemical oxygen demand (COD) and total ammoniacal nitrogen (TAN) concentrations in the leachate fluctuated, with the parameters decreasing as the leachate was diluted due to the replenishment with the CSTR effluent (Figure 1a and Figure 2a, respectively). The Organic Loading Rate (OLR) was 2.5–3.2 gCOD L_CSTR_^−1^ d^−1^ at the beginning of each cycle and decreased to 0.5−1 gCOD L_CSTR_^−1^ d^−1^ towards the end of the cycle. The Hydraulic Retention Time (HRT) was 15.6 days. A comparison of the COD concentration in the leachates shows that zeolite does not affect the organic load of the leachate, an observation that is compatible with the results of a previous work [22]. Since zeolite interacts with cations such as ammonia, it was not anticipated to affect the organic matter leaching.

On the other hand, the TAN concentration was higher in the leachate of the control experiment. It seems that the presence of zeolite successfully retained 200–800 mg TAN L^−1^. Moreover, the TAN concentration level increased from cycle to cycle in both LBRs since little water was added when the LBRs were fed with CM mixture to compensate for the saturation of the bed with humidity.

Figure 1b and Figure 2b demonstrate the changes in COD and TAN concentrations in the effluent of the CSTR, which was fed with the leachate of the LBR. The COD concentration inside the CSTR (Figure S1) was higher than in the effluent, as the effluent was withdrawn from the upper part of the reactor after the stirring had ceased for an hour. During the experiment, there was a decline in the COD due to the decrease in solids and soluble COD. A comparison of COD concentration between the zeolite and control experiment reveals that the COD in the control experiment was unstable. The increases in the effluent COD coincided, indicating that soluble compounds, such as VFAs, were temporarily accumulated, which raised the organic matter in the control CSTR.

The TAN concentration in the CSTR’s leachate and effluent was observed to increase, which indicates an accumulation of TAN. Figure 1b shows that the rate of increase in TAN concentration in the CSTR’s effluent in the system with the zeolite was lower (14.0 mg L^−1^ d^−1^) than in the control (17.3 mg L^−1^ d^−1^). The linear trend lines also support this observation.

**Figure 3:**
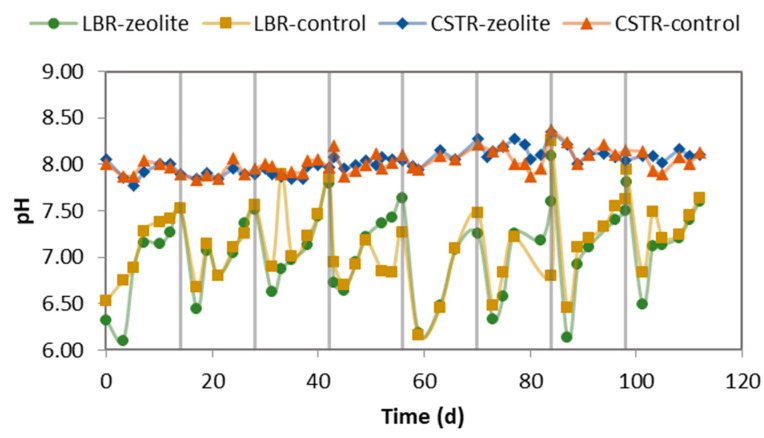
pH in the CSTR’s effluent and LBR’s leachate. The vertical lines indicate when the LBR was emptied and filled with fresh CM mixture.

The pH level of the leachate started off low at the beginning of each cycle but gradually increased until the end of the cycle. This trend is shown in Figure 3. After filling it with the CM mixture, we added approximately 90 mL of tap water to the LBR to ensure that the CM bed was fully saturated with moisture. This addition of water decreased the initial pH. However, as the leachate recirculated in the LBR bed, the pH increased, and the TAN was washed from the CM bed. Furthermore, the effluent of the CSTR also contributed to the gradual increase in pH at the end of the cycle. The pH in the CSTR stayed within a narrow range of values, around 8, with a slight tendency to increase. Upon comparing the two systems, we found no significant differences.

**Figure 4:**
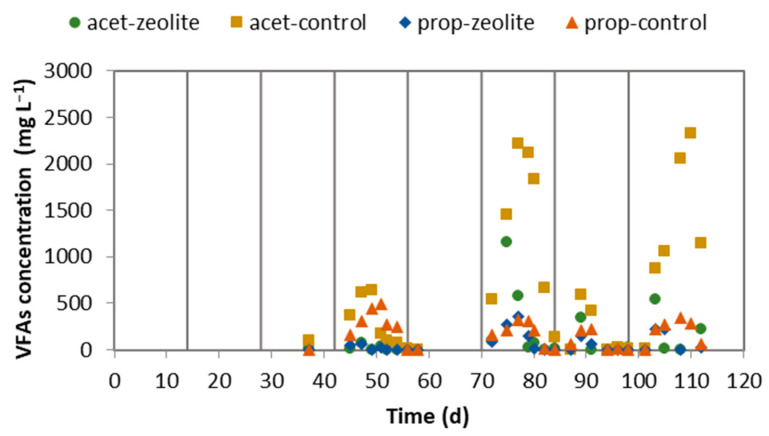
Acetate and propionate concentrations in the effluent of the CSTRs. The vertical lines indicate when the LBR was emptied and filled with fresh CM mixture.

The pH level is decreased by the concentration of VFAs and increased by the TAN concentration. The TAN concentration in the CSTR of the control system was higher, and the VFA concentration peaked at higher values than in the zeolite system (as seen in Figure 4), particularly in the last three cycles. As a result, when the VFA decreasing effect on the pH prevailed over the TAN increasing effect on the pH, the pH decreased, but this happened temporarily (coinciding with the VFA peaks). The control system had a significant accumulation of acetate concentration during the sixth and eighth cycles, reaching 2200–2300 mg L^−1^. In contrast, the acetate concentration in the zeolite system peaked below 1100 mg L^−1^. During the sixth cycle, the CSTR feeding stopped to reduce the acetate concentration. On the other hand, the zeolite system continued to operate without any intervention. The concentration of propionate remained below 500 mg L^−1^ in both systems.

**Figure 5:**
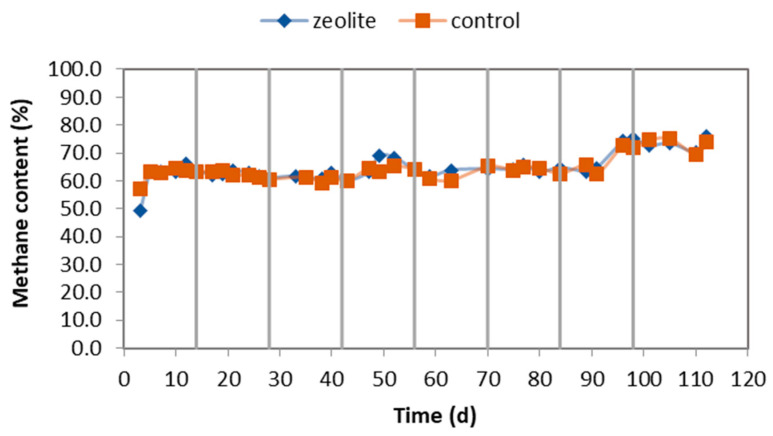
Methane percentage in biogas in each cycle in both systems CSTR. The vertical lines indicate when the LBR was emptied and filled with fresh CM mixture.

**Figure 6:**
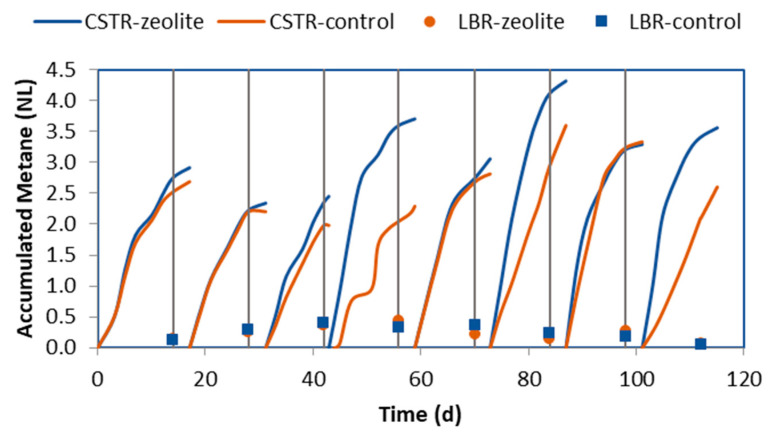
Methane accumulation in each cycle in both systems: CSTR (lines) and the LBR (symbols). The vertical lines indicate when the LBR was emptied and filled with fresh CM mixture.

Biogas with 60–65% methane content was produced by the CSTRs. Towards the end of the experiment, this increased to 70–75% (as shown in Figure 5). As TAN accumulated, the pH increased, leading to an increase in the methane percentage. However, the methane production from the CSTR in the control system (Figure 6) was lower than that of the zeolite system since the biogas produced was lower. Significant differences were observed in the third, fourth, sixth, and eighth cycles (as shown in Figure 6). The vertical lines in Figure 6 indicate the LBRs refilling with fresh CM mixture. The methane production in the CSTRs continues to increase after the vertical lines because, at this point, the CSTRs were fed with the leachate that was removed from the LBRs before they were emptied. The LBRs also produced biogas with 20–40% methane content and up to 14% hydrogen in some cases. The total methane produced in the LBRs at the end of each cycle is represented by symbols in Figure 6. The LBRs contributed much less to the total methane produced by the systems, and there were no significant differences in this contribution between the two systems.

**Figure 7:**
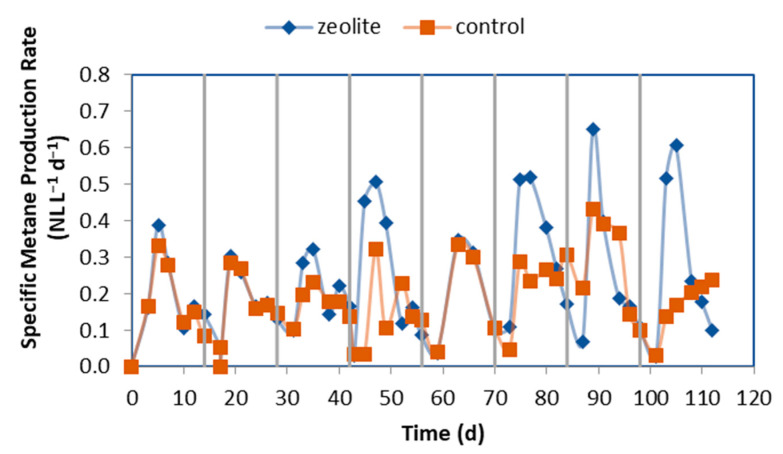
Specific methane production rate in each cycle in the CSTRs of both systems. The vertical lines indicate when the LBR was emptied and filled with fresh CM mixture.

During each cycle, the production of methane increased to its maximum point due to the addition of fresh organic matter to the leachate after feeding the LBR with CM, as shown in Figure 7. However, as the incoming leaching became less concentrated in organic matter, the rate of methane production quickly decreased to below 0.05 NL L_CSTR_^−1^ d^−1^), which proved that the SRT of 14 d was sufficient. In the zeolite system, the methane production rate peaked early in each cycle and then decreased sharply. In contrast, the control experiment showed a lower methane production rate, with a slow increase observed later in each cycle, indicating that the reactor was under stress.

Both systems showed similar responses during the initial cycles, as they were still affected by the inoculum of the CSTRs. However, significant differences in methane production regarding accumulation, rate, and yield were observed from the fourth cycle and onwards. The yield was calculated by dividing the accumulated methane produced in both CSTR and LBR per cycle by the VS of the CM added in the LBR at the beginning of each cycle, and it was found that the system with zeolite produced a higher yield (Table 1). Although in the fifth and seventh cycles, the yield of the control system was close to that of the zeolite system, each cycle with a lower yield in the control system was followed by a cycle where the yield reached that of the zeolite system. However, overall, the lower performance of the control system was significant, with a total yield (since the fourth cycle) of 0.142 NL CH_4_ gVS_in_^−1^ compared to 0.171 NL CH_4_ gVS_in_^−1^ estimated for the zeolite system when considering the total methane produced since the fourth cycle divided by the total amount of VS of CM fed in the system during this period.

### 2.2. Microbial Composition and Dynamics

**Figure 8:**
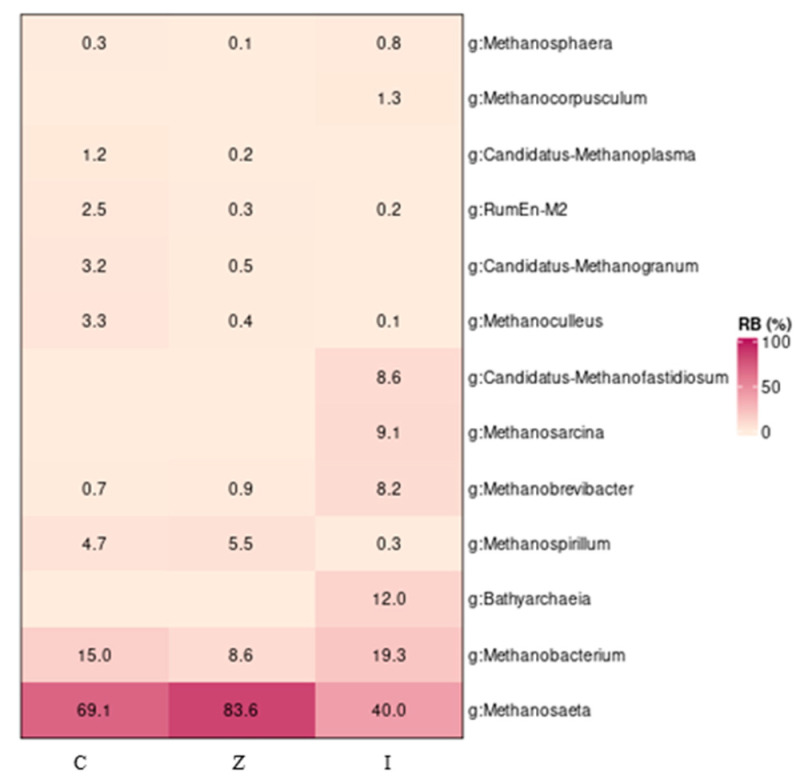
Relative abundance and diversity of the archaeal community revealed by sequencing of the 16S rRNA gene in samples from the CSTR fed on the leachate from the LBR amended with zeolite (Z) or pebble (C) as well as from the inoculum (I).

A total of 169,342 archaeal sequences were obtained across the nine samples (three biological replicates per sample—inoculum; I and zeolite; Z, control; C), with an average number of 18,815 sequences per site. These sequences were filtered (99,311 total reads and 11,034 reads per sample) and distributed between 18 different OTUs at 97% identity. In the investigated system, the archaeal representatives were less diverse than those of bacteria. Halobacterota clearly dominated the archaeal community at the phylum level, followed by Euryarchaeota in all the samples (Figure S2). The genera *Methanosaeta* and *Methanocterium* were present at a relative abundance (RA) of more than 40% and 8.6%, respectively, within this dataset (Figure 8). Especially in the control and zeolite samples, the OTUs affiliated with those two genera exceeded 85% of the total reads. It was interesting to observe that the genera *Bathyarchaea* (RA: 12%), *Methanosarcina* (RA: 9.1%), *Candidatus*-Methanofastidiosum (RA: 8.6%), and *Methanocorpusculum* (RA: 1.3%) were present only in the inoculum samples. At the same time, the genera *Candidatus*-Methanogranum (RA: 0.5–3.2%) and *Candidatus*-Methanoplasma (RA: 0.2–1.2%) were detected only in the control and zeolite samples (Figure 8).

**Figure 9:**
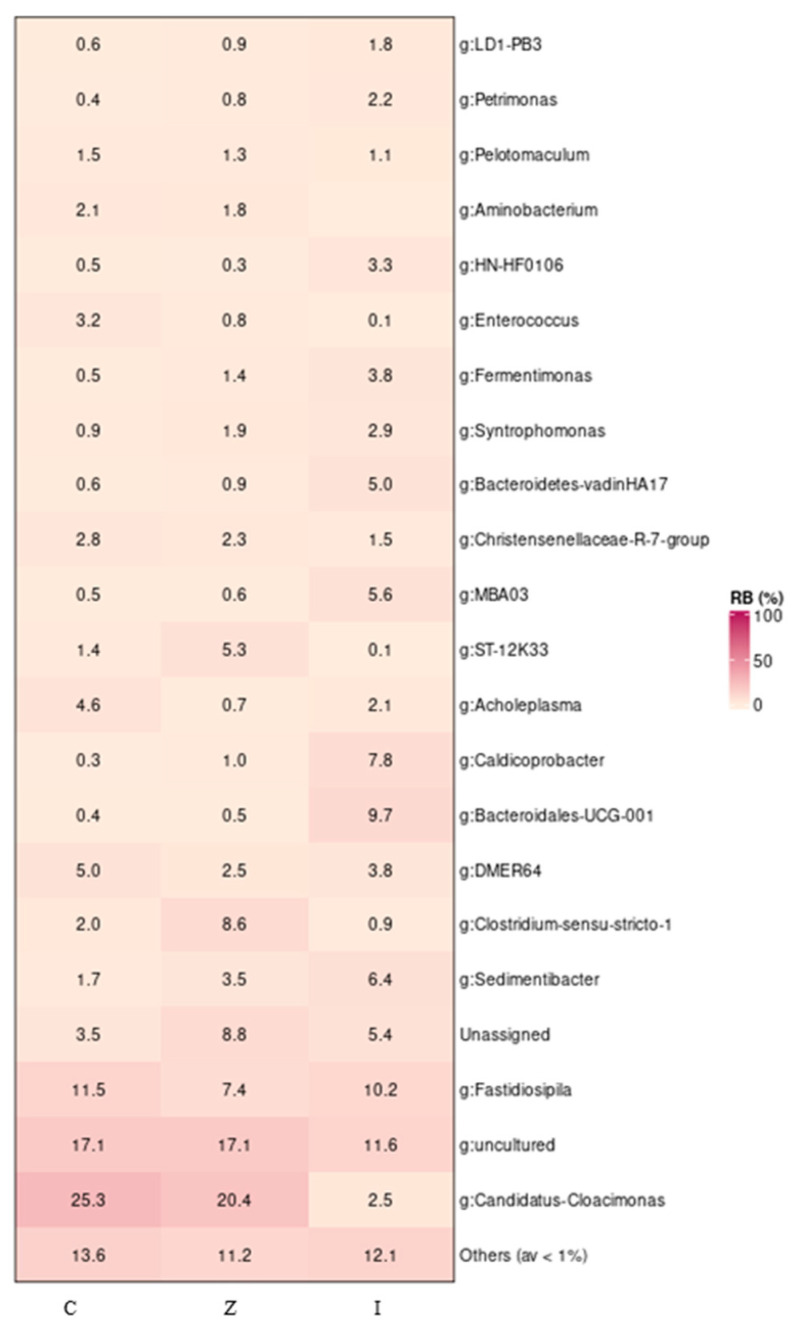
Relative abundance and diversity of the bacterial community revealed by sequencing of the 16S rRNA gene in samples from the CSTR fed on the leachate from the LBR amended with zeolite (Z) or pebble (C) as well as from the inoculum (I).

Bacterial community composition and diversity of the samples from the CSTR fed on the leachate from the LBR amended with zeolite (Z) or pebble (C), as well as from the inoculum samples (I), were investigated by sequencing the V3–V4 region of the 16S rRNA gene producing a total of 127,946 reads. After filtering low-quality sequences, 71,898 reads were clustered into OTUs at a 97% identity threshold with an average of 7988 reads/sample. A set of 159 distinct OTUs were present at a relative abundance of over 0.1% within the dataset. At the phylum level, the vast majority of the reads were affiliated with Firmicutes (RA: 40–53.2%) followed by Bacteroidota (RA: 22.9–27.3%) (Figure S3). Notably, phylum Cloacimonadota (previously Cloacimonetes) was abundant in the zeolite and control datasets with RA 20.4% and 25.2%, respectively, but did not exceed 2.5% in the inoculum samples. Cloacimonadota was dominated by *Candidatus*—Cloacimonas, which is associated with syntrophic amino acid and propionate degradation [23] and is present in many anaerobic digesters (Figure 9).

Bacterial community composition in the inoculum samples included higher levels of the following genera: *Sedimentibacter*, *Bacteroidales*-UCG-001, *Caldicoprobacter*, MBA03, *Bacteroidetes*-vadinHA17, *Fermentimonas*, HN-HFO106, *Petrimonas* and LD1-PB3 compared to samples taken from the CSTRs at the end of the experiment (C and Z). The bacterial consortium in C was dominated by *Candidatus*-Cloacimonas (RA: 25.3%), followed by uncultured representatives (RA: 17.1%) and *Fastidiosipila* (RA:11.5%). *Candidatus*-Cloacimonas (RA: 20.4%) was also the predominant bacterium genus in Z, followed by uncultured representatives, unassigned (RA: 17.1–8.8%, respectively) and *Clostridium* sensu stricto 1 (RA: 8.6%). The genus *Aminobacterium* was detected only in the CSTRs’ samples, with a stable percentage of around 2% (Figure 9).

Overall, the bacterial community within the investigated system had a significantly higher species richness (ACE) than that within the archaeal consortium (Figure S4). However, among the samples, the bacterial or archaeal richness was similar (Figure S4). Regarding the dynamics in archaeal diversity, similar patterns were observed among communities in the inoculum and the CSTRs samples (C and Z) (Figure S5). The PERMANOVA test indicated no significant difference between the samples (*p*-value > 0.05; Figure S6A). In contrast, significantly different bacterial communities were observed between the inoculum and the CSTRs’ samples (C and Z) (*p*-value < 0.05; Figure S6B). This means that only the bacterial profile of the inoculum was changed compared to the samples taken from the CSTRs at the end of the experiment, and the archaeal community remained stable with no significant differences.

## 3. Discussion

In a previous study, we found that the readily leachable organic matter of CM yielded more methane when leaching occurred in the presence of zeolite [22]. The biochemical methane potential (BMP) of the zeolite-assisted leachate from two samples of CM was 0.263 ± 0.027 NLCH_4_ g COD_leachate_^−1^ and 0.342 ± 0.013 NLCH_4_ g COD_leachate_^−1^, respectively. In contrast, the BMP of the leachate produced in the control experiments was 0.219 ± 0.008 NLCH_4_ g COD_leachate_^−1^ and 0.319 ± 0.008 NLCH_4_ g COD_leachate_^−1^, respectively. Physicochemical analysis of the leachates demonstrated that zeolite reduced the TAN concentration by 15% on average, while it did not affect the COD concentration. These observations are compatible with the leaching experiments performed in a previous study [22] investigating the characteristics of the leachates produced in LBRs amended with zeolite or pebbles (control) without the effect of microbial degradation. It is widely known that zeolite, when added to a reactor, promotes the production of biogas by aiding in the conversion of organic matter into methane due to TAN adsorption via ion exchange, which is possible since zeolite contains Na^+^, Ca^2+^, and Mg^2+^ cations in its crystalline structure. [19]. However, some studies have found that the increase in methane yield cannot be solely attributed to the adsorption of TAN or ion exchange on zeolite, as there was only a slight reduction in TAN in the presence of zeolite [15]. The present work adds to these findings by using zeolite in the LBR, rather than the CSTR, where most methane is produced. The beneficial effect of zeolite was observed through the leachate generated from the mixture of cow manure and zeolite. This leachate may have carried fragments of zeolite or other compounds, such as cations exchanged for ammonia adsorption, into the CSTR, offering stability in the process and supporting the findings of Spyridonidis et al. [22].

An LBR connected to a second reactor to treat the leachate operates in a batch-like manner because LBRs are loaded only once. However, a continuous mode of operation can be achieved by employing multiple LBRs that are loaded successively, allowing others to complete an entire cycle of operation before being emptied and reloaded. For instance, Nizami et al. [20] set up six leach beds that were emptied and fed sequentially every 7 or 5 days in series, resulting in a smoother OLR in the second stage, where methanogenesis occurred. Similarly, in our previous work, we used this concept to equalize the OLR in the second stage by using three LBRs connected to one CSTR, with each LBR being emptied and fed every 5 days [7]. As a result, the OLR varied in a narrower range (0.5–1 gCOD L^−1^ d^−1^) than in the present work.

The AD of CM anaerobic digestion in CSTR-type reactors results in a higher methane performance than the performance achieved in this study. Zhang et al. [24] estimated that CM could produce 0.338–0.419 L gVS_in_^−1^ of biogas with 60–65% CH_4_ at an HRT of 20–45 d. However, Reyes et al. [25] emphasized the importance of treating the liquid stream coming from CM through leaching rather than treating the heterogeneous, high-solid mixture of CM. The LBR technology can produce leachate from high solid content wastes, such as manures. Two-stage dry AD comprising an LBR for the first stage can produce methane at 0.080–0.166 L gVS_in_^−1^ in cases of manures [21,26], even if the AN concentration is 500 mg L^−1^, far below the inhibitory level [26]. In the present study, the LBR-CSTR coupling achieved a yield of 0.171 NL L gVS_in_^−1^, corroborating the performance of 0.17 NL gVS_in_^−1^ achieved when operating three series LBRs connected with one CSTR [7]. Both studies demonstrate a lower yield than what can be obtained in a CSTR. However, LBR is a dry AD system and offers the advantage of slight water addition only to replenish the humidity of the saturated CM mixture when the LBR is emptied. It also generates the leachate, which is further treated and produces biogas. In this way, the introduction of inert material mingled with CM into a homogenous digester (such as CSTR) is avoided, and all serious operational problems associated with the presence of inert materials (such as accumulation and reduction of digester’s volume, equipment damage, etc.) are prevented. The LBR was completely emptied before being fed again. The methane yield could be improved if some CM was kept as inoculum from the previous cycle.

The accumulation of non-biodegradable compounds, such as AN, can occur in two-stage dry AD with internal recirculation, which can be inhibitory at some level [21]. The study showed that TAN had an accumulative trend, but the accumulation rate was lower in the case of the zeolite-assisted system. Similarly, the electrical conductivity (EC) increased during the experiment in both the leachate and the CSTR effluent (Figure S7). However, the EC of the CSTR effluent treating the leachate from the CM/zeolite mixture was 25.5 mS cm^−1^ at the end of the eighth cycle compared to 29.5 mS cm^−1^ in the control system. To prevent the build-up of AN and EC, the occasional removal of effluent and the addition of tap water could be applied.

In this study, the microbial community compositions and dynamics were studied in the inoculum compared to the samples taken from the CSTRs (Z, C) using amplicon sequencing of the 16S rRNA gene with the technology of Illumina MiSeq. The bacterial community within the investigated system had a significantly higher species richness (ACE) than the archaeal consortium. Moreover, the bacterial diversity pattern differed in Z and C compared to the inoculum. In contrast, the archaeal community remained stable with no significant differences.

The inoculum was taken from a biogas plant operating with cow and chicken manures (40–50% and 15–20%, respectively) permanently and triticale/corn silages, olive mill/cheese wastewaters, molasses/glycerin as supplementary feedstocks when available [27]. The archaeal taxonomic composition of the inoculum was dominated by the genera *Methanosaeta*, *Methanobacterium,* and *Bathyarchaeia*, all with vast metabolic capabilities and widespread in anoxic environments. *Methanosaeta* species are speculated to be the predominant CH_4_ producers on Earth [28]. It is well reported that archaeal communities from agricultural waste and sewage sludge digesters are dominated by the obligate acetolactic genus *Methanosaeta* [29]. Although *Methanosaeta* have previously been thought to be restricted to acetate as a substrate for methane production, they have the potential to use carbon dioxide for methane production [30]. *Methanobacterium*, despite the name, belongs not to the bacterial domain but the archaeal domain and can use formate to reduce methane; others live exclusively by reducing carbon dioxide with hydrogen. They are ubiquitous in some hot, low-oxygen environments, such as anaerobic digestors, wastewater, and hot springs [31]. Methanogenic Archaea are micro-organisms that are capable of producing methane gas, and the typical methanogens include species of *Methanobacterium*, *Methanosarcina*, and *Methanosaeta*. Methanogens serve an important role in the global carbon cycle, completing the conversion of organic carbon into methane gas. This process is syntropic, meaning that the products of the metabolic activities of other micro-organisms are used as the substrates for methanogenesis. During this process, communities of resident bacteria degrade biopolymers into alcohols and fatty acids, and the fatty acids, subsequently, into acetate and carbon dioxide. Some bacteria in these communities also produce hydrogen as a metabolic end product. Methanogens then utilize these products to generate methane. The most widely used substrate for methanogenesis is acetate, which accounts for two thirds of the methane generated in these environments [32]. *Bathyarchaeia* are characterized by their numerical dominance and metabolic versatility in the anoxic deep biosphere. To date, pure cultures of *Bathyarchaeia* have not been successfully isolated [33]. Genomic analyses suggest that *Bathyarchaeia* lead an acetyl-CoA-centered heterotrophic lifestyle with the potential for acetogenesis, methane metabolism, and sulfur reduction. *Bathyarchaeia* also encompass a variety of genes encoding carbohydrate-active enzymes and, thus, likely can unitize various carbohydrates and lignin [34]. The diverse metabolic potential of *Bathyarchaeia* contributes to their predominance in sedimentary environments, rendering them essential players in the global carbon cycle [35]. The main adaptive strategy for methanogens in the environment is to exist as part of syntropic communities or consortia with Bacteria [32]. The various complex anaerobic reactions that lead to methane formation are, to a large extent, performed through syntrophy between bacteria and methanogenic archaea. These syntrophic relationships provide the methanogens with their substrates and remove metabolic products from the acid-forming bacteria [36].

Regarding the bacterial taxonomic composition of the inoculum, higher levels of *Sedimentibacter* (pyruvate and amino acid consumers) [37] *Bacteroidales*-UCG-001 (involved in hydrolysis, acidogenesis, and acetogenesis steps) [38] *Caldicoprobacter* (thermophilic sugar consumer) [39], MBA03 (thermophilic carbohydrate consumers) [40], *Bacteroidetes*-vadinHA17, *Fermentimonas* (acidogens) [41], HN-HFO106, *Petrimonas*, and LD1-PB3 (potentially involved in hydrolysis, fermentation, and acetogenesis) [42] were observed compared to the CSTR samples.

There was a significant difference in the bacterial community of the CSTRs between the samples at the end of the experiment and the inoculum. However, no significant difference was observed between the control and zeolite experiments. Cloacimonadota dominated by *Candidatus*-Cloacimonas were present in both C and Z samples at higher RA (25.3% and 20.4%, respectively) than in inoculum (2.5%). They are found primarily in engineered waste treatment environments (such as anaerobic digesters and landfill leachates) [43], but new studies have discovered them in natural habitats [44]. They are capable of deriving sugars from complex carbohydrates, fermenting sugars to acetate, and promoting propionate oxidation to acetate. In addition to sugar fermentation, they have also been correlated with amino acid [23] and lipid [45] fermentation. They are frequently characterized as syntrophic bacteria living with hydrogen consumers such as methanogens or sulfate reducers [44].

In this study, *Aminobacter* was found only in the CSTR samples and is believed to be a potential syntrophic acetate oxidizer that has a positive correlation with hydrogenotrophic methanogens [46]. On the other hand, the acetoclastic *Methanosaeta* increased from 40% in the inoculum to 69.1% in the control experiment and even more in the zeolite experiment (83.6%). Although zeolite has been known to enhance hydrogenotrophic methanogenesis in the presence of ammonia at the expense of acetoclastic methanogenesis [47], this does not seem to be the case here, as acetoclastic archaea tend to dominate. The higher level of acetoclastic *Methanosaeta* in the zeolite experiment coincides with lower acetate concentration as well as the higher stability of the CSTR compared to the control experiment. Although the archaeal community did not differ significantly throughout the experiment, some genera like *Bathyarchaea*, *Methanosarcina*, *Candidatus*-Methanofastidiosum, and *Methanocorpusculum* were found only in the inoculum samples. On the other hand, the genera *Candidatus*-Methanogranum and *Candidatus*-Methanoplasma were detected only in the CSTR samples.

It should be noted that while the microbial analysis cannot provide quantitative conclusions, the VSS concentration could be used to indicate the total concentration of microorganisms. This is because the feeding leachates did not contain solids and had almost identical COD concentrations (as can be observed in Figure 1a). The results showed that the VSS concentration in the CSTR of the zeolite system was higher than in the control system (as seen in Figure S8).

It is important to stress that in the zeolite experiment, the zeolite did not come into direct contact with the microbial community of the CSTR. Instead, it was present in the LBR, which produced the leachate fed to the CSTR. Therefore, any positive impact observed was probably induced by chemical agents from the zeolite’s contact with the leachate, which then entered the CSTR. This may explain the slight, but not statistically significant, differences observed in the control and zeolite experiments regarding both the archaeal and bacterial communities.

## 4. Materials and Methods

### 4.1. CM and Zeolite Origin and Characteristics

The CM used in the experiment was obtained from a broiler farm in the broader area of Xanthi, located in the Region of Eastern Macedonia and Thrace, Greece. The CM was collected in plastic bags and immediately taken to the Wastewater Management and Treatment Technologies Laboratory at the Department of Environmental Engineering, Democritus University of Thrace. Before the experiment, the CM was pre-treated to remove any feathers and large stalks, which were collected along with the bedding material of broilers. The CM was then stored in a refrigerator at 4 °C. Two batches of CM were collected at different periods with slightly different characteristics. These differences were likely due to the manual collection process at different times. The characteristics of each batch used in the experimental period are presented in Table 2.

The natural zeolite used in this study was obtained from MEC Energy, Mining construction industry & trade Co in Ankara, Turkey. Its particle size was between 2–2.5 mm. The chemical composition of the zeolite is presented in Table S1 and consists of 90% Clinoptilolite, 5% Feldspar, and 0–4% Cristobalite. To remove any moisture, the zeolite was dried at 105 °C for 24 h before each use. The pebbles were taken from a local river shore. They were chosen as a substitute for the zeolite in the control system since they are continuously washed off with nonsaline water (such as river water) and have no ion exchange properties such as zeolite. In this sense, zeolite could be regarded as an “inert” material compared to zeolite.

### 4.2. Experimental Set-Up and Operation

**Figure 10:**
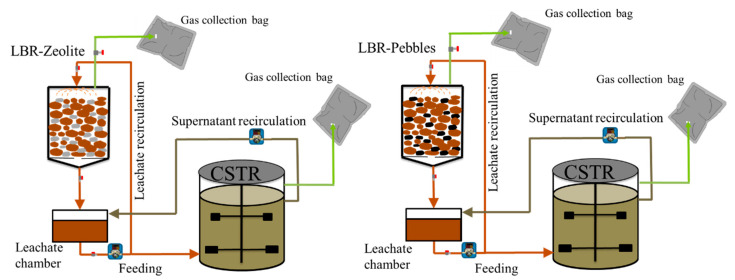
Experimental set-up of two LBR-CSTR systems.

Two identical set-ups were created, each consisting of one LBR connected to one CSTR and operated separately, as shown in Figure 10. The LBRs were made of glass, with an internal diameter of 4.5 cm and a bed height of 30 cm. Each LBR had a total volume of 250 mL, with a waste bed volume of 220 mL (CM bed volume). Hot water was recirculated through plastic flexible tubes wrapped around the LBRs to maintain a constant temperature of 38 ± 1 °C. The first LBR was filled with CM (25.18 ± 0.1 gVS) and zeolite at a 0.8 g zeolite g^−1^VS ratio, as per previous works [7,27].

In this work, wood chips (0.28 g wood chips g^−1^VS) were also added to prevent clogging. The second LBR was filled with the same mixture but with pebbles instead of zeolite and was used as a control system. Every 14 days, the LBRs were emptied and refilled with a new mixture (CM: 25.18 ± 0.1 gVS, zeolite or pebbles: 20.14 ± 0.8 g and wood chips: 7.05 ± 0.28 g). During the first 42 days, the LBRs were loaded with CM from Batch 1 and, after that, with CM from Batch 2 (Table 2).

Each LBR was connected to a 250 mL working volume chamber for collecting leachate. After filling the LBRs, 240 mL of tap water were added to soak the CM/zeolite/straw mixture and remove atmospheric air. About 90 mL of the added water were absorbed by the CM/zeolite/straw mixture, and the remaining water (approximately 150 mL) was allowed to drain in the chamber, making up the leachate. Macronutrients and trace elements were added to the collection tank, as per previous studies, to provide the necessary nutrients for the process [48,49]. To maintain a constant volume of leachate in the chamber, it was replenished with tap water after sampling. A bag filled with a mixture of CO_2_/N_2_ was placed in the leachate chamber. The leachate was recirculated over the CM mixture bed at a rate of 2.64 L L^−1^LBR d^−1^ to ensure sufficient solid–liquid contact, as per previous works [22].

Each CSTR (1.25 L of total volume and 1 L of working volume) was fed manually with 150 mL of the leachate produced from the LBR three times per week (average feeding flowrate 64.30 mL d^−1^). Therefore, the hydraulic retention time (HRT) of the CSTR was 15.6 d. Prior to feeding, the stirrer was stopped to allow for solid settling in the CSTR. After an hour, 150 mL of the supernatant was removed manually and returned to the LBR. By doing so, the addition of solids that would cause clogging was avoided while simultaneously supplying the LBR with digestate to enhance the microbial processes and replenish the leachate that was removed from the LBR. The CSTRs were initially inoculated with biomass (11.47 gVSS L^−1^) from a full-scale biogas plant that treated cow manure and other feedstocks [27]. The CSTRs were placed in an incubator at a temperature of 37 ± 1 °C and were mixed using a magnetic stirrer.

### 4.3. Analytical Methods

Chemical analysis was conducted on CM samples, as well as leachate and CSTR samples, measuring Chemical Oxygen Demand (COD), Total Ammonia Nitrogen (TAN), Total Solids (TS), Volatile Solids (VS), Total Suspended Solids (TSS), Volatile Suspended Solids (VSS), and Total Kjeldahl Nitrogen (TKN), according to Standard Methods [50]. pH and electrical conductivity were measured using a pH meter (HANNA HI 83141 HANNA Instruments, Woonsocket, RI, H∏A) and a conductivity meter (Crison, CM 35, Crison Instruments, SA, Barcelona, Spain). Gas chromatography (GC) (Shimadzu GC-2014, Shimadzu, Kyoto, Japan), equipped with a flame ionization detector and a capillary FFAP column, was used to measure volatile fatty acid (VFA) concentrations [7]. Biogas produced by the LBRs and CSTR was collected in aluminum bags, and its volume was determined using the water displacement method. The biogas composition was analyzed using a GC (Shimadzu GC-2014) equipped with a thermal conductivity detector (TCD) [7]. Biogas and methane volumes were expressed at STP (standard temperature and pressure) conditions.

### 4.4. Microbial Analysis

#### 4.4.1. DNA Extraction and 16S rDNA Amplification

Samples (three replicates) were collected from the CSTR and fed on the leachate from the LBR, which was amended with zeolite or pebble and the inoculum. They were stored at −20 °C until further processing. The DNA extraction from each sample (1 g) was performed using a commercial Kit (Higher Purity Soil DNA Isolation Kit, Canvax, Valladolid, Spain). Extraction was based on DNA’s ability to bind silica in the presence of high concentrations of chaotropic salts as guanidinium thiocyanate. Samples were rapidly and efficiently lysed by bead beating. The sample DNA was then bound to the surface of a silica membrane that was inside the spin column and washed, and the bound DNA was then desorbed from the surface of the membrane. The quantity and quality of the extracted DNAs were analyzed by a Q5000 micro-volume UV-Vis spectrophotometer (Quawell Technology, San Jose, CA, USA), and DNAs were stored in Eppendorf tubes at −20 °C until PCRs.

16S rDNA amplification was performed in triplicates using KAPA Taq Polymerase kit (KAPA BioSystems, Merck, Deutschland, Germany). The hypervariable V3–V4 region of the bacterial 16S rRNA gene was amplified using MiSeq universal primers 341F and 805R [51]. Two recent studies that evaluated the best performing primer pair based on bacterial taxa diversity coverage (Klindworth et al., 2013; Thijs et al., 2017) [51,52] recommend the primer pair 341F-805R, which amplifies the V3–V4 region, over other primer pairs. In more detail, in the Klindworth et al. [51] study, 512 primer pairs were tested in silico against the SILVA v108 database (376,437 sequences) for amplification of archaeal and bacterial sequences. This primer pair was shown to be the least biased among 512 primer pairs evaluated in silico for bacterial amplification and was experimentally shown to give a taxonomic composition similar to that of shotgun metagenomics. The study by Thijs et al. [52] used both in silico, as well laboratory experiments, to access the best primer pair. The final volume of the reaction was 25 μL containing KAPA Taq Buffer (10×) at a final concentration of 1×, dNTP mix solution (200 μM each), forward and reverse primer solutions (0.4 μM), 0.5 U of KAPA Taq DNA polymerase (5 U/μL), ≤100 ng from the template DNA solution, and sterile deionized water. The amplification protocol included a 3 min incubation at 95 °C followed by 35 cycles of 95 °C for 30 s, 55 °C for 30 s, and 72 °C for 1 min, and a final 3 min extension at 72 °C. Targeted variable regions (V3–V5) of the archaeal 16S rRNA gene were amplified with the MiSeq primer pair 519F and 915R [51]. This set of primers (519F/915R) offers the highest resolution for archaebacterial diversity analysis [51,53,54,55]. The mastermix was performed as mentioned above, and the protocol was as follows: 3 min denaturation at 95 °C; 35 cycles of 95 °C for 20 s, 57 °C for 15 s and 72 °C for 45 s, and 3 min of final elongation step at 72 °C. Negative and positive controls were always performed at the same time.

The resulting amplicons were then separated in a 1.5% (*w*/*v*) agarose gel in TAE buffer (40 mM Tris-acetate, 1 mM EDTA), while the product, with a length of approximately 550 bp was visualized in Bio-Rad’s Gel Doc XR+ system (Bio-Rad, Hercules, CA, USA). The purification of the products from unincorporated primers and nucleotides was performed with a 20% PEG, 2.5 M NaCl solution, and subsequent centrifugation at 14,000× *g* for 20 min [56]. The precipitate was washed twice with 125 µL of a 70% *v*/*v* ethanol solution and centrifuged at 14,000× *g* for 10 min. The precipitates were dried and suspended in 15 µL of sterile deionized water. Their concentration was estimated with a Q5000 micro-volume UV–Vis spectrophotometer (Quawell Technology, San Jose, CA, USA).

#### 4.4.2. Library Preparation and Illumina MiSeq Sequencing

The next-generation sequencing workflow contained the following steps: library preparation, sequencing, and data analysis. The purified PCR products, diluted up to 10 ng/µL, were used as templates for further amplification. This step attaches dual indices and Illumina sequencing adapters in a 50 µL volume containing 5 µL KAPA Taq buffer 10×, 0.4 µL dNTPs (25 mM), 0.2 µL of KAPA Taq DNA polymerase, 5 µL of the forward index primer (10 µM), 5 µL of the reverse index primer (10 µM), 2 µL of the amplicon diluted up to 10 ng/µL, and 32.4 µL of sterile deionized water. The combinatorial use of index primers resulted in unique samples pooled and sequenced on one Illumina MiSeq run. Libraries were again purified using the NucleoMag NGS (next-generation sequencing) clean-up and size selection kit (Macherey-Nagel, Düren, Germany), according to the manufacturer’s recommendations. The barcoded amplicon libraries were combined in equal concentrations (8 nM) into a single pool according to their Nanodrop quantification measurement, and the sequencing was performed by Macrogen (Seoul, Republic of Korea) using a 2 × 300 bp pair-end kit on a MiSeq platform. The raw reads were deposited into the NCBI Sequence Read Archive database (accession number: PRJNA1101897).

#### 4.4.3. Bioinformatics Analysis

Raw sequencing reads were demultiplexed, converted to FASTQ, and the Illumina adapters were trimmed using Illumina standard algorithms. The bioinformatics analysis was performed using a combination of USEARCH v.11 [57] and Qiime2 distribution 2019.1 [58]. Unassembled reads and reads outside the expected range were discarded. The quality of assembled sequences was improved using -fastq_filter, followed by the -fastx_uniques command to detect unique read sequences and their frequencies. Then, assembled reads were clustered into operational taxonomic units (OTUs) at 97% sequence similarities using the cluster_otus command based on the UPARSE algorithm [59]. Crosstalk errors were identified and filtered using the -uncross command based on the UNCROSS2 algorithm [60]. The extremely rare OTUs (<0.001% of total sequences across all samples) were discarded using otutab_trim command. Taxonomy was assigned to the representative sequences of the OTUs in Qiime2 based on the BLAST+ algorithm [61] and searching against SILVA 138 release database [62] with a 0.91% identity as a cutoff.

Alpha diversity reflects the diversity of individual samples and was calculated based on vegan R package (Version 2.2-0) [63] and plotted using ggplot2 R package (Version 3.5.1) [64]. ACE (abundance-based coverage estimators) is an indicator of species richness (total number of species in a sample) that is sensitive to rare OTUs (singletons and doubletons) [65]. High values indicate a high diversity. The graphical results are presented in boxplots with experimental groups on the x-axis and ACE on the y-axis annotated with significance labels (bars with asterisks, * *p* < 0.05, ** *p* < 0.01 and *** *p* < 0.001) above the boxplots. Statistical comparisons of the indices between the groups are calculated using analysis of variance (ANOVA), followed by Tukey’s honestly significant difference (HSD).

Beta diversity quantifies the distances between different microbial profiles described by the OTUs table, which allows to link the overall taxonomic diversity pattern to the experimental features. The visualization of the multidimensional distance matrix in a space of two dimensions was performed by Canonical Analysis of Principal coordinate (CAP) [66]. Statistically significant differences between samples were identified with permutational multivariate analysis of variance (PERMANOVA) [67] using 999 permutations and Monte Carlo tests. CAP was performed on PRIMER version 6 and PERMANOVA+ for PRIMER routines [68,69]. The non-parametric permutation test PERMANOVA was used to assess the significant clustering between the groups. Moreover, a pairwise comparison of the beta diversity metric was conducted, and the results were plotted in a heatmap graph. The PERMANOVA test indicates that no significant difference was detected (similar microbial communities) when *p*-value is higher than 0.05 (yellow color).

## 5. Conclusions

The dry AD of CM took place in a two-stage configuration consisting of an LBR coupled with a CSTR, with the CSTR’s supernatant recirculated to the LBR. The addition of zeolite (at a ratio of 0.8 g per g of volatile solids (VS)) to the chicken manure bed improved the methane yield by 20% compared to the control experiment without zeolite. The methane yield reached 0.171 NL g^−1^VS. The recirculation of the CSTR’s supernatant to the LBR and the minimal water addition while feeding the LBR resulted in a gradual ammonia build-up, meaning that dilution with fresh water would be necessary in the long term. However, this was not necessary during the 120d of the experimental run since ammonia was kept below 3000 mg L^−1^. The ammonia build-up occurred at a lower rate in the system with zeolite; however, the difference in the ammonia concentration between the two systems was less than 500–600 mg L^−1^ towards the end of the experiment. Feeding the CSTR with the zeolite-amended leachate resulted in a more stable system with lower volatile fatty acid accumulation and a higher methane production rate, compared to the control experiment.

Although the archaeal profiles of both CSTRs (zeolite and control) towards the end of the experiment did not differ significantly (*p* > 0.05), there was a higher RA of acetoclastic *Methanosaeta* in the zeolite experiment than in the control. In contrast, the opposite occurred with the hydrogenotrophic *Methanobacterium*. The bacterial community of the inoculum evolved during the experiment and resulted in a statistically significant different profile (*p* < 0.05), which was predominated by syntrophic *Candidatus*-Cloacimonas in both systems. However, the archaeal community evolved in similar patterns during the experiment (*p* > 0.05).

## Figures and Tables

**Table 1:** Methane yield estimated for each cycle.

Cycle No	Methane Yield (NL CH_4_ gVS_in_^−1^)	
	Zeolite	Control
4	0.178	0.113
5	0.150	0.146
6	0.204	0.174
7	0.159	0.157
8	0.165	0.120

**Table 2:** Chicken manure characteristics.

CM	Parameter			
	TS (g kg^−1^FM)	VS (g kg^−1^FM)	COD (g kg^−1^VS)	TKN (g kg^−1^VS)
1st batch	828 ± 0.8	677 ± 2.76	1250 ± 78	59 ± 4.4
2nd batch	619 ± 9.2	502 ± 9.3	1307 ± 70	63 ± 2.8

FM: Fresh Matter, COD: Chemical Oxygen Demand.

## Data Availability

The raw reads were deposited into the NCBI Sequence Read Archive database (accession number: PRJNA1101897).

## Supplementary Materials

The following supporting information can be downloaded at: https://www.mdpi.com/article/10.3390/molecules29112568/s1, Table S1: Physicochemical properties of zeolite; Figure S1: Total and soluble COD concentration in the CSTR treating the leachate of the LBR filled with zeolite (zeol) or pebbles (cont). The vertical lines indicate when the LBR was emptied and filled with fresh CM mixture.; Figure S2: Heatmap of archaeal relative abundances at phylum level. SAMPLES: C: LBR with pebbles (control), Ζ: LBR amended with zeolite, I: inoculum samples.; Figure S3: Heatmap of bacterial relative abundances at phylum level. SAMPLES: C: LBR with pebbles (control), Ζ: LBR amended with zeolite, I: inoculum samples.; Figure S4: Species richness (ACE) within the examined samples: C: LBR with pebbles (control), Ζ: LBR amended with zeolite, I: inoculum. (A) Archaea (B) Bacteria.; Figure S5: Canonical Analysis of Principal Coordinate (CAP) of microbial communities (A) Archaea (B) Bacteria. Samples: C: LBR with pebbles (control), Ζ: LBR amended with zeolite, I: inoculum.; Figure S6: PERMANOVA pairwise comparison between samples (C: LBR with pebbles (control), Ζ: LBR amended with zeolite, I: inoculum) (A) Archaea (B) Bacteria.; Figure S7: Electrical Conductivity (EC) of the (a) leachate and (b) CSTR effluent during the experiment. The vertical lines indicate when the LBR was emptied and filled with fresh CM mixture; Figure S8: VSS concentration in the CSTR treating the leachate of the LBR filled with zeolite or pebbles. The vertical lines indicate when the LBR was emptied and filled with fresh CM mixture.
