# Supplementary material for: Enhancement of Biodegradability of Chicken Manure via the Addition of Zeolite in a Two-Stage Dry Anaerobic Digestion Configuration
Source: Molecules. 2024 May 30;29(11):2568. doi: 10.3390/molecules29112568 (PMC11173769; doi:10.3390/molecules29112568)
Supplement: Supplementary file 1 [file molecules-29-02568-s001.zip › molecules-3011101-supplementary.pdf]

Supplementary material

Enhancement of biodegradability of chicken manure via the addition of zeolite in a two-stage dry anaerobic digestion configuration.

Achilleas Kalogiannis[1], Ioanna A. Vasiliadou[1,2,], Athanasios Tsiamis[3], Ioannis Galiatsatos[3], Panagiota Stathopoulou[3], George Tsiamis[3] and Katerina Stamatelatou[1,*]

[1] Department of Environmental Engineering, Democritus University of Thrace, Vas. Sofias 12, GR-67132 Xanthi, Greece; achkalog@env.duth.gr (A.K.); ivasiliadou@uowm.gr (I.A.V.)
[2] Department of Chemical Engineering, University of Western Macedonia, GR-50100 Kozani, Greece
[3] Laboratory of Systems Microbiology and Applied Genomics, Department of Sustainable Agriculture,
University of Patras, GR-30131 Agrinio, Greece; etsiamis9@gmail.com (A.T.); jgalia96@gmail.com (I.G.); panstath@upatras.gr (P.S.); gtsiamis@upatras.gr (G.T.)
* Correspondence: astamat@env.duth.gr; Tel.: +30-2541079315

Table S1: Physicochemical properties of zeolite.

Chemical composition											
**Chemical compound**	Loss on Ignition	Al_2O_3	CaO	Fe_2O_3	K_2O	MgO	MnO	Na_2O	P_2O_5	SiO_3	TiO
**Percentage (%)**	6.25	13.2	2.0	1.4	3.5	1.1	<0.1	0.3	<0.1	71.9	0.1

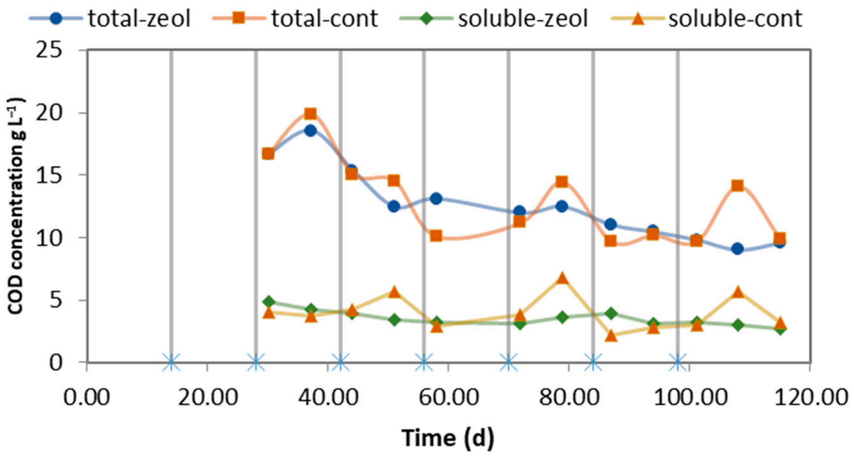

Figure S1: Total and soluble COD concentration in the CSTR treating the leachate of the LBR filled with zeolite (zeol) or pebbles (cont). The vertical lines indicate when the LBR was emptied and filled with fresh CM mixture.

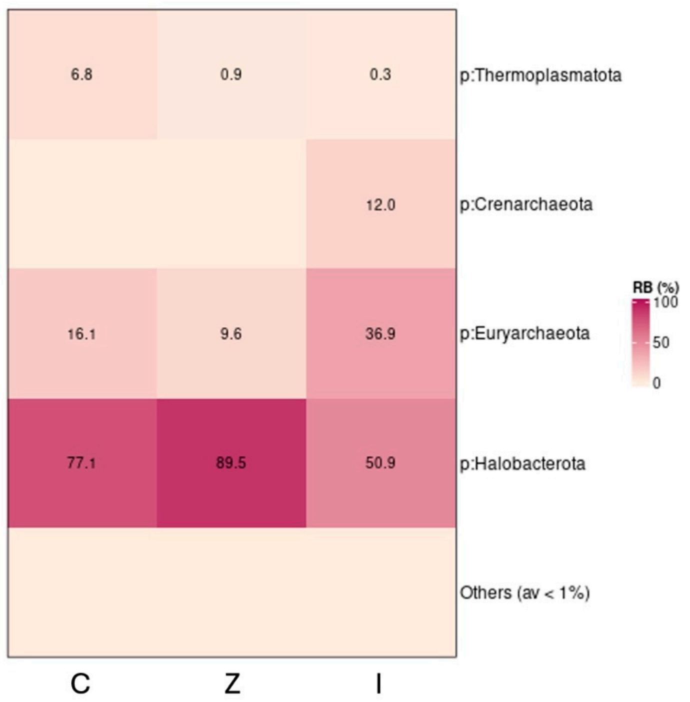

Figure S2: Heatmap of archaeal relative abundances at phylum level. SAMPLES: C: LBR with pebbles (control), Z: LBR amended with zeolite, I: inoculum samples.

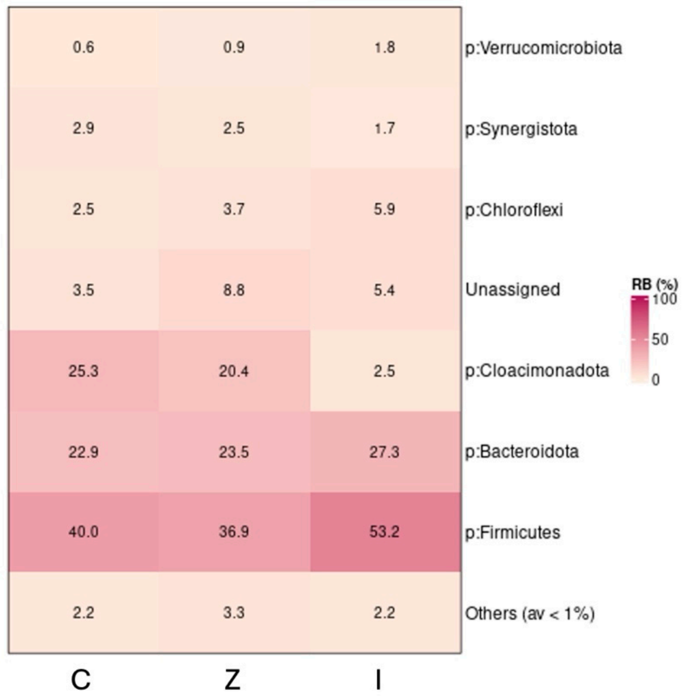

Figure S3: Heatmap of bacterial relative abundances at phylum level. SAMPLES: C: LBR with pebbles (control), Z: LBR amended with zeolite, I: inoculum samples.

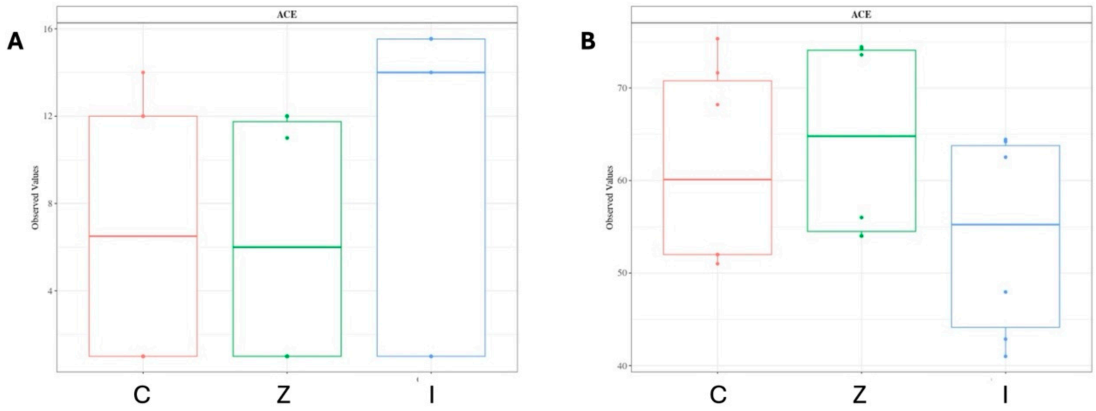

Figure S4: Species richness (ACE) within the examined samples: C: LBR with pebbles (control), Z: LBR amended with zeolite, I: inoculum. (A) Archaea (B) Bacteria.

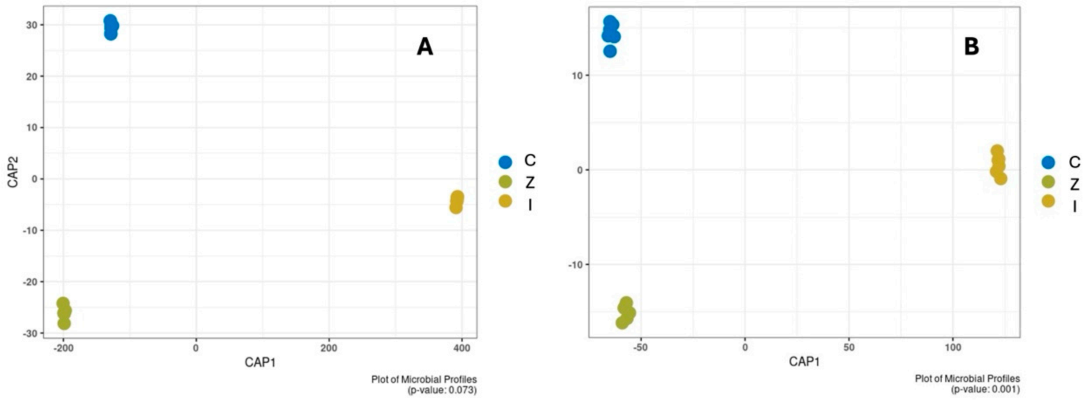

Figure S5: Canonical Analysis of Principal Coordinate (CAP) of microbial communities (A) Archaea (B) Bacteria.

Samples: C: LBR with pebbles (control), Z: LBR amended with zeolite, I: inoculum.

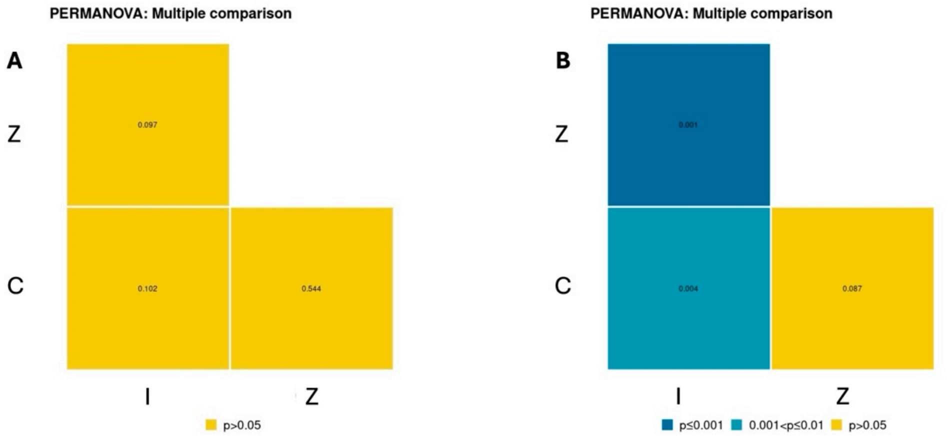

Figure S6: PERMANOVA pairwise comparison between samples (C: LBR with pebbles (control), Z: LBR amended with zeolite, I: inoculum) (A) Archaea (B) Bacteria.

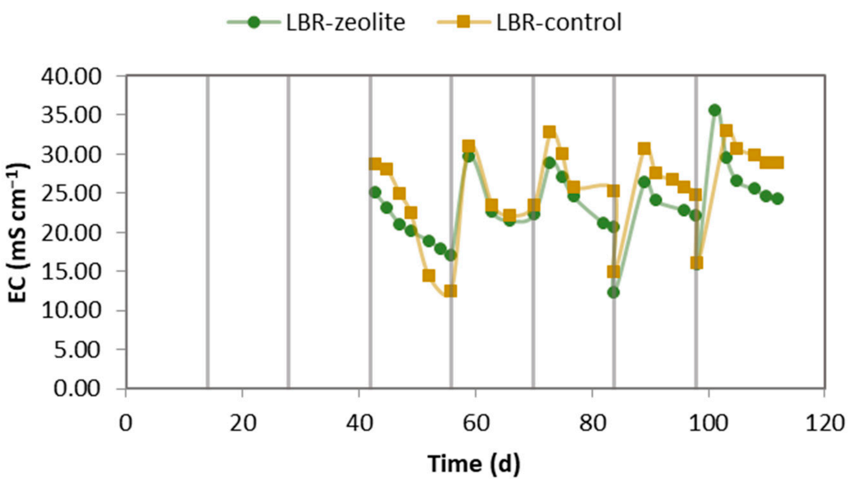

(a)

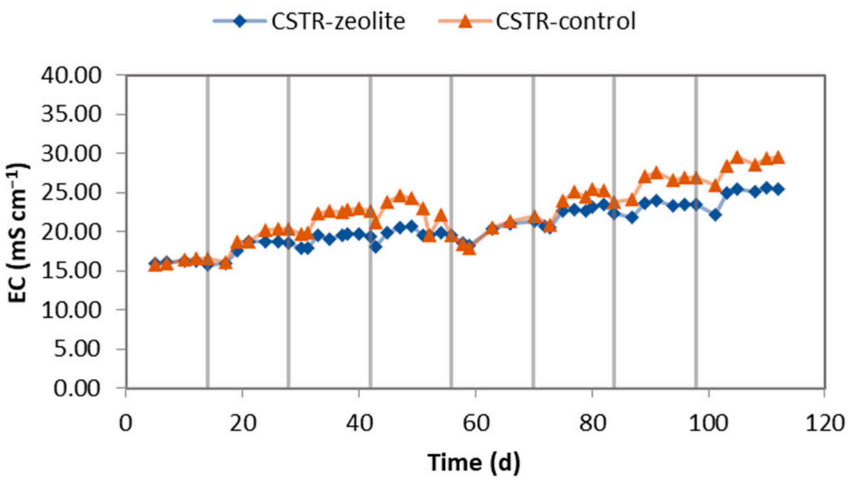

(b)

Figure S7: Electrical Conductivity (EC) of the (a) leachate and (b) CSTR effluent during the experiment. The vertical lines indicate when the LBR was emptied and filled with fresh CM mixture.

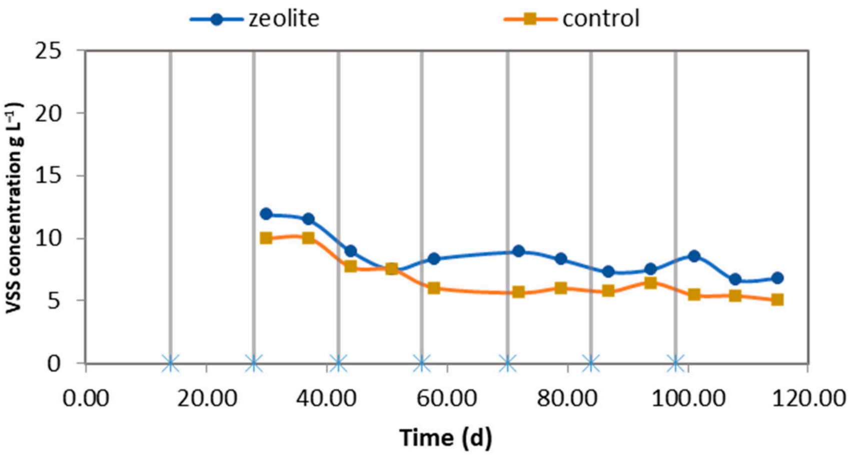

Figure S8: VSS concentration in the CSTR treating the leachate of the LBR filled with zeolite or pebbles. The vertical lines indicate when the LBR was emptied and filled with fresh CM mixture.
